# Injectable Hyaluronic Acid-*co*-Gelatin Cryogels for Tissue-Engineering Applications

**DOI:** 10.3390/ma11081374

**Published:** 2018-08-07

**Authors:** Mahboobeh Rezaeeyazdi, Thibault Colombani, Adnan Memic, Sidi A. Bencherif

**Affiliations:** 1 Department of Chemical Engineering, Northeastern University, Boston, MA 02115, USA; rezaeeyazdi.m@husky.neu.edu (M.R.); t.colombani@northeastern.edu (T.C.); amemic@gmail.com (A.M.); 2 Center of Nanotechnology, King Abdulaziz University, Jeddah, Makkah 21589, Saudi Arabia; 3 Department of Bioengineering, Northeastern University, Boston, MA 02115, USA; 4 Harvard John A. Paulson School of Engineering and Applied Sciences, Harvard University, Cambridge, MA 02138, USA; 5 Biomechanics and Bioengineering (BMBI), UTC CNRS UMR 7338, University of Technology of Compiègne, Sorbonne University, 60203 Compiègne, France

**Keywords:** injectable, hyaluronic acid, gelatin, scaffold, cryogel, tissue engineering

## Abstract

Polymeric scaffolds such as hydrogels can be engineered to restore, maintain, or improve impaired tissues and organs. However, most hydrogels require surgical implantation that can cause several complications such as infection and damage to adjacent tissues. Therefore, developing minimally invasive strategies is of critical importance for these purposes. Herein, we developed several injectable cryogels made out of hyaluronic acid and gelatin for tissue-engineering applications. The physicochemical properties of hyaluronic acid combined with the intrinsic cell-adhesion properties of gelatin can provide suitable physical support for the attachment, survival, and spreading of cells. The physical characteristics of pure gelatin cryogels, such as mechanics and injectability, were enhanced once copolymerized with hyaluronic acid. Reciprocally, the adhesion of 3T3 cells cultured in hyaluronic acid cryogels was enhanced when formulated with gelatin. Furthermore, cryogels had a minimal effect on bone marrow dendritic cell activation, suggesting their cytocompatibility. Finally, in vitro studies revealed that copolymerizing gelatin with hyaluronic acid did not significantly alter their respective intrinsic biological properties. These findings suggest that hyaluronic acid-*co*-gelatin cryogels combined the favorable inherent properties of each biopolymer, providing a mechanically robust, cell-responsive, macroporous, and injectable platform for tissue-engineering applications.

## 1. Introduction

The aim of tissue and regenerative engineering is to replace damaged, degenerated, or defective tissues using scaffolds, cells, and signaling molecules [1,2,3,4,5,6]. Polymeric scaffolds can be engineered using hydrogels that are made up of highly hydrophilic and crosslinked three-dimensional (3D) polymer networks [7,8,9]. However, traditional hydrogels often exhibit a nanoporous network that can limit cell motility, proliferation, and survival, as well as poor mechanical flexibility [10,11,12,13,14,15]. On the other hand, cryogels, a unique class of hydrogels prepared via cryopolymerization, exhibit large tunable interconnected macropores, high elasticity, and flexibility [15,16,17,18,19]. The ability to fabricate macroporous 3D cryogel matrices provides a promising strategy for promoting cell–biomaterial and cell–cell interactions [20,21,22]. A variety of extracellular matrix (ECM) components and other biopolymers can be used to generate bioinspired cryogels for tissue engineering applications [17,19,23,24,25].

Hyaluronic acid (HA), a naturally occurring polysaccharide, is a major ECM component and an important structural element in various tissues. HA has been recognized for its role in the formation of the cardiac jelly during heart morphogenesis [26,27,28]. Furthermore, HA can play an important role in angiogenesis and wound healing [12,29]. The polysaccharide is composed of d-glucuronic acid and D-*N*-acetyl glucosamine repeating units that can be degraded by hyaluronidase enzyme [30,31]. Hydrogel scaffolds mimicking various tissues have been made using HA [31,32]. These biomaterials have been shown to exhibit a rapid ability to absorb body fluids, a high moisture content, a long-term water-retention ratio, and good flexibility [33]. These properties are important for various tissue-engineering applications; for example, HA can play an important role in the maintenance of joint lubrication, or in the hydration and moisturization of tissues [34]. However, HA is unable to promote cell adhesion and attachment across a number of mammalian cells [32,35,36] even though it is generally considered to be non-immunogenic and non-thrombogenic [31,37].

To improve the cell-adhesive properties of HA, several groups have suggested coupling HA with gelatin [29,36,38,39]. Gelatin is a naturally derived polymer enabling cell attachment due to their intrinsic integrin-binding domains [40]. Gelatin is obtained from hydrolyzed collagen, the most abundant protein in the human body [10]. This protein has been extensively utilized in tissue engineering, especially as mechanical support for various types of cells, including neural, cartilage, and liver cells, as well as a matrix for the cryopreservation of cells [36,41,42,43,44,45]. The presence of matrix metalloproteinase (MMP)-sensitive peptide sequences makes gelatin easily degradable, and thus an attractive biomaterial [45,46].

Recently, several strategies have been investigated to enhance the properties of hydrogel scaffolds, including combining various biopolymers [16,17,47]. Several groups have combined HA and gelatin with other biopolymers to generate hydrogels with tunable biophysical properties [18,20,24,32,35,48,49]. These gels were fabricated using various crosslinking mechanisms, including Michael-type addition reaction [50,51], polycondensation, click reactions [52,53], Schiff’s base formation [54,55], and free-radical polymerization [32,48,56,57,58,59]. These conventional hydrogels typically require invasive implantations for their placement into the body [15]. Due to the trauma associated with surgical implantations, a number of approaches have been developed to deliver hydrogels in a minimally invasive manner [18,60].

HA and gelatin have been combined for the design of injectable hydrogels. For example, thiolated gelatin, HA, and poly(ethylene glycol) diacrylate exhibit sol-to-gel transitions and were administrated into the body for osteochondral repair [39,61]. Similarly, partially oxidized gelatin and HA have been used for applications in nucleus pulposus support [37]. These systems consist of injecting a liquid into the body, which is allowed to solidify in situ. Unfortunately, these approaches have several drawbacks. For instance, slow-reacting precursors are likely to leak from the injection site and potentially cause inflammation [62]. Furthermore, if the gelation is too fast, it can cause premature gel formation and clog the needle during injection, but it can also alter the physical properties of the gel itself [63]. One way to alleviate these issues associated with traditional approaches is to fabricate hydrogels that are fully polymerized and yet flowable through a needle. Recently, injectable cryogels have gained tremendous momentum in the field, as they are syringe-injectable and fully polymerized scaffolds retaining their integrity and properties once introduced into the body. Cryogels display improved mechanical stability when compared to traditional hydrogels, as their unique macrostructure provides increased strength for the gels to undergo compaction and full recovery at the injection site [13,26,31,35]. Additionally, cryogels have been utilized for a variety of biomedical applications, including tissue engineering [44,45,46].

Free-radical polymerization is the most common crosslinking method used during cryogel fabrication [20,31,40]. In order to chemically crosslink HA and gelatin through free-radical polymerization, pendant methacrylate groups need to be covalently incorporated into the polymer chains [48,64,65]. In this study, we synthesized methacrylate derivatives of HA (HAGM) and gelatin (MA-gelatin) to be used as precursors for the fabrication of injectable and biomimetic cryogels. Under various fabrication conditions, cryogels were prepared by polymerizing methacrylated HA and gelatin, individually or in combination. We investigated the physical properties of the resulting cryogels, including swelling ratio, pore size, pore interconnectivity, mechanical behavior, and syringe injectability. Furthermore, we evaluated the influence of fabrication temperature on the physical properties of cryogels as well as the retention of their biological properties (interactions of polymers with their corresponding antibodies) upon cryopolymerization. Additionally, we showed that the cryogels can undergo injection through a 16-gauge needle while retaining scaffold structure and integrity. Ultimately, cryogels fabricated in this study can be tuned to exhibit improved biological and cell-adhesive properties with minimal cytotoxicity. Taken together, these naturally derived and bioinspired HAGM-*co*-MA-gelatin (HA-*co*-Gelatin) cryogels show great potential for applications in tissue engineering and regenerative medicine.

## 2. Materials and Methods

### 2.1. Synthesis of HAGM and Gelatin (MA-Gelatin)

To prepare HA and gelatin amenable to cryogelation, pendant methacrylate groups were first introduced into the main chains of these polymers. HAGM was prepared by grafting methacrylate groups to hyaluronic acid [32,35,66]. A total of 1 gram of hyaluronic acid (Sigma-Aldrich, St. Louis, MO, USA) was dissolved in 200 mL phosphate buffered saline (PBS, Sigma-Aldrich, pH 7.4) and subsequently mixed with 67 mL of dimethylformamide (Sigma-Aldrich), 13.3 g of glycidyl methacrylate (Sigma-Aldrich), and 6.7 g of triethylamine (Sigma-Aldrich). After 10 days of reaction, the solution was precipitated in an excess of acetone, filtered, and dried overnight in a vacuum oven. Amine terminated HAGM was prepared by grafting adipic acid dihydrazide to HAGM. Briefly, 1 g HAGM was dissolved in 100 mL 2-(*N*-Morpholino) ethanesulfonic acid (MES, pH 5.5). Adipic acid dihydrazide (4 g, Sigma-Aldrich) and EDC (90 mg) were subsequently added to the mixture while stirring at room temperature (RT). After 4 h of reaction, the modified polymer was precipitated in an excess acetone, filtered, and dried in a vacuum oven at RT. Amine terminated HAGM was then labeled with NHS-Rhodamine. Aminated HAGM (1 g) was dissolved in 10 mL of sodium bicarbonate buffer (NaHCO_3_, pH 8.5). NHS-Rhodamine (5/6-carboxy-tetramethyl-rhodamine succinimidyl ester, ThermoFisher, Waltham, MA, USA) (10 mg) was subsequently added to the mixture while stirring at RT. After an overnight reaction, the product was precipitated in excess of acetone, filtered, and dried in a vacuum oven overnight and stored at −20 °C until further use.

MA-gelatin was prepared by reacting a solution of type-A gelatin from porcine skin (Sigma-Aldrich) in deionized water (d-H_2_O) and dimethyl sulfoxide with a 60-fold molar excess of methacrylic anhydride in the presence of triethylamine [48]. Briefly, 50 mL of DMSO was added to 100 mL of d-H_2_O containing 8 g of gelatin at 60 °C. A total of 40 g of methacrylic anhydride and 3 g of triethylamine were subsequently incorporated within the mixture while stirring vigorously at 60 °C for 3 days. Next, the modified polymer was precipitated in an excess of methanol, filtered, and then dried in a vacuum oven overnight at RT. The polymer was further dissolved in d-H_2_O, dialyzed for 3 days against d-H_2_O, lyophilized, and stored at −20 °C until further use. Rhodamine-labeled MA-gelatin was synthesized following the same protocol used for aminated HAGM in this study. Briefly, MA-gelatin (1 g) was dissolved in 10 mL of sodium bicarbonate buffer (NaHCO_3_, pH 8.5). NHS-Rhodamine (ThermoFisher) (10 mg) was subsequently added to the mixture while stirring at RT. After an overnight reaction, the Rhodamine-labeled MA-gelatin was obtained by precipitating the reaction solution in excess methanol. The product was then dried in a vacuum oven overnight and stored at −20 °C until further use.

### 2.2. Chemical Characterization of Polymers and Cryogels by ^1^H NMR

^1^H NMR analysis was conducted to calculate the degree of methacrylation and assess qualitatively vinyl group consumption during cryogelation using a Varian Inova-500 NMR spectrometer. Deuterium oxide (D_2_O) was used as solvent, and the concentration of the modified polymers was kept at 1% (*w*/*v*). For chemical characterization of the cryogels, cryogelation was induced directly in an NMR tube. One milliliter of the prepolymer solution containing the initiator was transferred into the NMR tube before cryogenic treatment at −20, −50, and −80 °C for 15 h. All ^1^H NMR spectra were obtained at RT, 15 Hz sample spinning, 45° tip angle for the observation pulse, and a 10 s recycle delay, for 128 scans. Peak values at 5.2 and 5.5 ppm for HAGM and at 5.4 and 5.7 ppm for MA-gelatin were correlated to the presence of methacrylated groups. Peak areas were integrated using ACD/Spectrus NMR analysis software and degrees of methacrylation for each polymer type were determined. The degree of methacrylation of HAGM was calculated from the ratio of the relative peak integration of the methylene protons and the peaks correlated to the protons of the main polymer chain [48,64]. For MA-gelatin, the degree of methacrylation, which is defined as the ratio of the number of amine groups functionalized with methacrylamide groups to the total number of amine groups present in gelatin prior to the reaction, can be determined by comparing the integrated intensity of the aromatic region, representing the concentration of gelatin, with the intensity of the double-bond region [48].

### 2.3. Preparation of HAGM (4% w/v), MA-Gelatin (4% w/v), and HA-co-Gelatin (2% HAGM + 2% MA-Gelatin, wt/v) Cryogels

Cryogels were synthesized by redox-induced free-radical cryopolymerization in d-H_2_O at three different subzero temperatures (−20, −50, and −80 °C) [20]. Biopolymers (individually or in combination) were dissolved in d-H_2_O to the final concentration of 4% (*w*/*v*) in the presence of 0.5% (*w*/*v*) ammonium persulfate (APS, Sigma-Aldrich) and 0.1% (*w*/*v*) tetramethylethylenediamine (TEMED, Sigma-Aldrich). The precooled prepolymer solutions at 4 °C were quickly poured into Teflon molds (4 mm × 4 mm × 1 mm), transferred to a freezer at a preset subzero temperature (−20, −50, or −80 °C), and allowed to cryopolymerize. After 15 h of reaction, the resulting cryogels (HAGM, MA-gelatin, or HA-*co*-Gelatin) were brought to RT and washed with deionized water.

### 2.4. Physical Characterization of Cryogels

Young’s moduli were determined using an Instron testing system (Instron 5944, Instron, Norwood, MA, USA). Cylindrical cryogels (6 mm diameter, 6 mm height) were dynamically deformed (at a constant rate) between two parallel plates for 10 cycles with a strain rate of 10% per minute. Compressive strain (mm) and load (N) were then measured at the 8th cycle using an Instron’s Bluehill 3 software (Instron, Norwood, MA, USA). Moduli were determined by obtaining the tangent of the slope of the linear region on the loading stress/strain curve. The gel cylinders were kept hydrated in PBS (pH 7.4) throughout the tests. The swelling ratio was determined using a conventional gravimetric procedure. To investigate the swelling ratio of each sample, cylindrical cryogels were prepared and immersed in PBS for 24 h prior to experiment. The equilibrium mass swelling ratio (QM) was calculated by dividing the mass of fully swollen cryogel by the mass of freeze-dried cryogel. The cryogels were washed in d-H_2_O prior to freeze-drying. To test degrees of pore interconnectivity, fully hydrated cryogels were first weighed on an analytical scale. Next, a Kimwipe was lightly applied to the scaffolds’ surfaces to wick away free water. The weight of partially dehydrated cryogels was recorded again. The degree of pore interconnectivity was calculated based on the amount of water wicked away divided by the initial total amount of water.

### 2.5. Injectability Test

Cuboid-shaped cryogels (4 mm × 4 mm × 1 mm) were suspended in 0.2 mL of PBS and successfully syringe-injected by means of a 16-gauge needle. Briefly, cryogels were positioned on the aperture of the needle and forced through the needle. The videos were taken using a Canon camera then mounted via Adobe Premiere Pro CC 2018.

### 2.6. Scanning Electron Microscopy (SEM)

For SEM, freeze-dried cryogels (4 mm × 4 mm × 1 mm) were mounted on the sample holder using carbon tape and sputter-coated with platinum/palladium up to 5 nm of thickness. Samples were then imaged using secondary electron detection on a Hitachi S-4800 scanning electron microscope (Hitachi High-Technology Corporation, Tokyo, Japan) while operating at 3 kV and 10 µA.

### 2.7. Biological Properties

The intrinsic biological functions (interactions of polymers with their corresponding antibodies) of HA and gelatin were evaluated via immunofluorescence assays (anti-CD44 and anti-gelatin antibodies, respectively) [67,68]. Briefly, cryogels were saturated for 1 h at RT with 1X PBS + 0.05% Tween-20 + 1% bovine serum albumin (BSA). Next, cryogels were incubated for 3 h at RT with either 10µg/mL of recombinant human CD44 His-tagged (CD44-3961H, Creative Biomart, Shirley, NY, USA) or anti-porcine gelatin anti-serum (GELP12-S, Alpha Diagnostic International, San Antonio, TX, USA) diluted 1:100 in 1X PBS + 0.05% Tween-20 + 1% BSA. Next, cryogels were treated for 1 h at RT in the dark with Alexa Fluor 488-conjugated 6x-His Tag monoclonal antibody (Fisher Scientific) or FITC-conjugated anti-rabbit IgG (Fc-region) (Alpha Diagnostic International), respectively. Cryogels were washed 3 times in 1X PBS + 0.05% Tween-20 between each step. Methacrylated alginate (MA-alginate) cryogels polymerized as previously described [20] were used as a negative control for these experiments.

### 2.8. In Vitro Cell Viability and Cytoskeleton Staining

Mouse Fibroblast cell line (NIH/3T3, CRL- 1658, ATCC, Rockville, MD, USA) was cultured in Dulbecco’s Modified Eagles Medium (DMEM) supplemented with 10% Fetal Bovine Serum (FBS, Sigma-Aldrich, St. Louis, MO, USA), 100 µg/mL penicillin (Fisher scientific, Hampton, NH, USA), and 100 µg/mL streptomycin (Fisher Scientific, Hampton, NH, USA) at 37 °C in a humidified 5% CO_2_/95% air-containing atmosphere. Prior to cell seeding, cryogels (4 mm × 4 mm × 1 mm) were treated with 70% ethanol for 30 min, then washed several times with sterile water. Cryogels were mechanically compressed on sterile gauze to partially remove pore water under sterile conditions before cell seeding. Ten microliters of cell suspension containing 2 × 10^5^ cells in a complete culture medium were added in a dropwise manner on top of each cuboid-shaped cryogel and incubated for 4 h. The cell-loaded cryogels were supplemented with 1 mL of fresh media for the extent of the experiment (37 °C in 5% CO_2_ environment). Cell distribution was noted to be homogeneous throughout the scaffold. Cell viability was determined via fixable dead cell assay. After 1 and 3 days of incubation, cells were treated with a far-red fixable dead cell staining according to manufacturer’s instructions (ViaQuant™, Genecopoeia, Rockville, MD, USA). Cells were then fixed with 4% formaldehyde solution (PFA, Sigma-Aldrich, St. Louis, MO, USA) for 30 min at RT and washed with PBS. Prior to microscopy, cells were permeabilized with PBS supplemented with 0.1% Triton X-100 (Sigma-Aldrich, St. Louis, MO, USA) for 5 min, then stained with DAPI (Sigma-Aldrich, St. Louis, MO, USA) and Alexa Fluor 488-phalloidin (Cell Signaling Technology, Danvers, MA, USA) according to manufacturer’s protocols. Confocal microscopy images were acquired using a Leica TCS SP5 X WLL Confocal Microscope (Buffalo Grove, IL, USA) and analyzed using ImageJ software (Version 1.52e, Bethesda, MD, USA). For each cryogel, 4 representative sections were analyzed. Percent viability was determined as the fraction viable cells to the total number of cells.

### 2.9. Generation of Bone Marrow-Derived Dendritic Cells (BMDC) and In Vitro Dendritic Cells (DCs) Activation Assays

Eight-week-old C57Bl/6 mice (The Jackson Laboratory, Bar Harbor, ME, USA) were housed in conventional conditions according to NIH guidelines. All animal experiments were performed in accordance with NIH recommendations, and approved by the DLAM ethics committee at Northeastern University. BMDC were extracted from C57BL/6 femur bone marrow as described by Lutz et al. [69], then cultured for 6 days in RPMI 1640 (Fisher Scientific) supplemented with 10% heat-inactivated FBS, 100 U/mL penicillin, 100 µg/mL streptomycin, 50 µM 2-mercaptoethanol (Fisher Scientific), and 20 ng/mL murine GM-CSF (Genscript, Piscataway, NJ, USA). To evaluate BMDC activation induced by different types of cryogels, one cuboid-shaped HAGM, MA-gelatin, or HA-*co*-Gelatin cryogel (4 mm × 4 mm × 1 mm) was incubated with BMDC in complete culture medium for 1 day (RPMI 1640 supplemented with 10% FBS and 1% penicillin-streptomycin). BMDCs maturation was then evaluated by flow cytometry (BD FACSCalibur DxP upgraded, Cytek Bioscience, Fremont, CA, USA) using the following fluorescent antibodies (Biolegend, San Diego, CA, USA): MHC II (M5/114.15.2, Rat IgG2b, PE/Cy7), CD86 (GL1, rat IgG2a, PE), CD11c (N418, hamster IgG, APC). Cytokine levels (IL-6, IL-12 and TNF-α) in the cell culture supernatant were analyzed by ELISA (IL12(p70)/TNF-α ELISA MAX Deluxe, BioLegend) according to the manufacturer’s instructions. Negative control consists of BMDCs cultured in media alone, while positive control consists of BMDCs incubated in media containing 100ng/mL of lipopolysaccharide (LPS).

### 2.10. Statistical Analysis

All values in the present study were expressed as mean ± SD. Statistical analyses were performed using GraphPad Prism Software (La Jolla, CA, USA). Significant differences between groups were analyzed by a Mann–Whitney test or a one-way analysis of variance (ANOVA). Differences were considered significant at *p* < 0.05.

## 3. Results

### 3.1. Polymer Synthesis and Characterization

**Figure 1:**
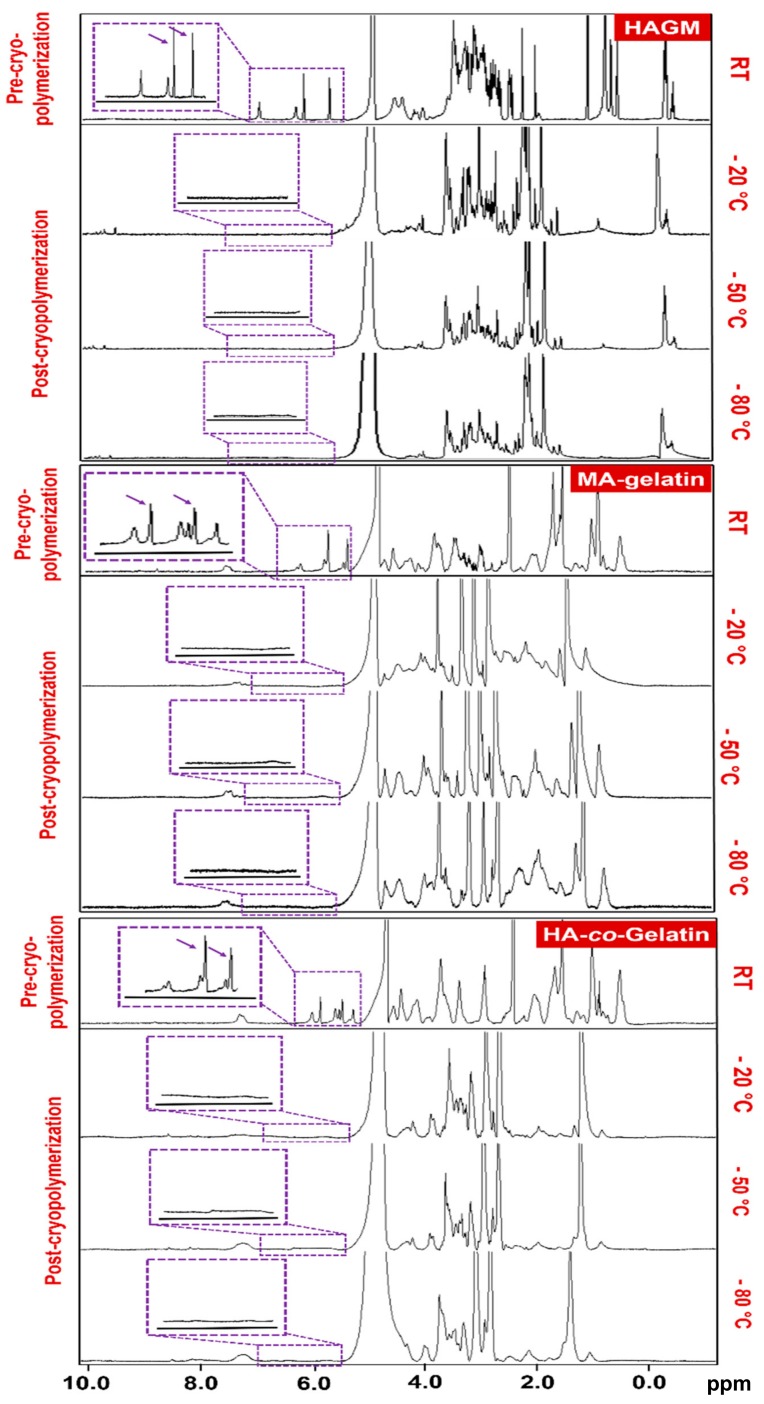
Chemical characterization of polymers by ^1^H NMR. ^1^H NMR spectra of methacrylate derivatives of hyaluronic acid (HAGM), MA-gelatin, and HA-*co*-Gelatin in D_2_O before and after cryopolymerization at various subzero temperatures (−20, −50 and −80 °C). Cryogelation was induced directly in an NMR tube. One milliliter of macromonomer solution containing the initiator system was transferred into the NMR tube before cryogenic treatment at each subzero temperature for 15 h. The vinylic peaks (between 5.5 and 6.5 ppm) disappeared after crosslinking.

As a precursor to the fabrication of cryogels, biopolymers were first functionalized with methacrylol moieties. For the synthesis of HAGM, ^1^H NMR spectrum confirmed the presence of vinyl methylene and methyl peaks at 5.5, 5.2 and 1.1 ppm, respectively. The newly formed peaks were the result of the reaction of hyaluronic acid with glycidyl methacrylate. Similarly, ^1^H NMR spectrum of MA-gelatin exhibited peaks at 5.8, 5.4 and 1.2 ppm after the reaction of gelatin with methacrylic anhydride (Figure 1A). HAGM and MA-gelatin macromonomers were found to have approximatively degrees of methacrylation of 31% and 25%, respectively. Figure 1B–D revealed that the methylene peaks disappeared after cryopolymerization across the three temperatures investigated, suggesting total consumption of the reactive pendant methacrylate groups (Figure S1).

### 3.2. Fabrication of HAGM, MA-Gelatin, and HA-co-Gelatin Cryogels

**Figure 2:**
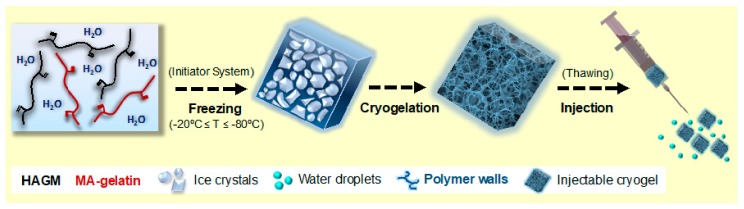
Schematic depicting the fabrication process of injectable HA-*co*-Gelatin cryogels. HAGM and/or MA-gelatin are dissolved in water containing an initiator system (APS and TEMED), and subsequently frozen at three different subzero temperatures (−20, −50 and −80 °C). The cryopolymerization and gelation of polymers occur around ice crystals, leaving interconnected voids behind upon melting. Rhodamine-labeled HAGM or MA-gelatin were incorporated only for microscopy imaging. Fabricated macroporous cryogels (4 mm × 4 mm × 1 mm) can then be easily and successfully syringe-injected through a 16-gauge hypodermic needle.

Cryogels were fabricated at three different subzero temperatures: −20, −50, and −80 °C [14,20,24]. During cryopolymerization, most of the water is frozen, and the dissolved solutes (biopolymers and initiator system) are concentrated in a nonfrozen liquid microphase. Free-radical cryopolymerization and gelation occur around ice crystals, used here as tunable porogens (Figure 2). After nearly a 15 h reaction, cryogels were thawed at RT and subsequently washed to remove unreacted polymeric precursors and initiators. This technique allows the fabrication of injectable 3D cryogel scaffolds with an interconnected macroporous architecture [20,31,40].

### 3.3. Physical Characterization

**Figure 3:**
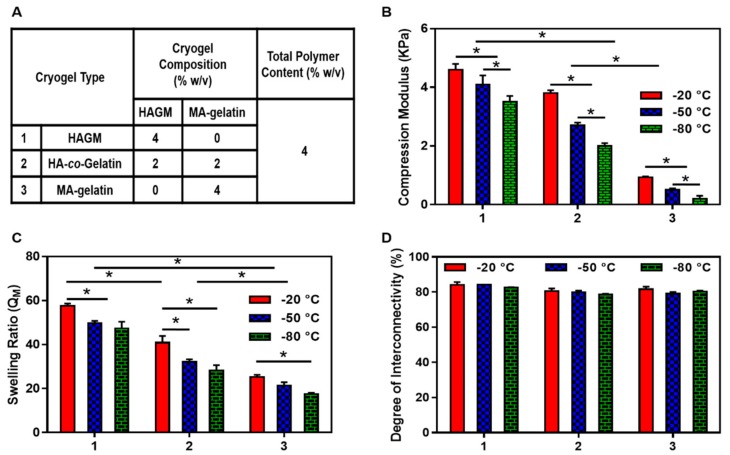
Physical characterization. (**A**) Cryogel type and composition; (**B**) compression moduli; (**C**) swelling ratios; and (**D**) degrees of pore interconnectivity of HAGM, MA-gelatin, and HA-*co*-Gelatin cryogels fabricated at −20, −50 and −80 °C. Values represent mean and SD (*n* = 5). Data were analyzed using one-way analysis of variance (ANOVA) (* *p* < 0.05).

The effects of the cryopolymerization temperature on the physical properties of cryogels with different compositions as stated in Figure 3A was investigated. Figure 3B shows the effect of the cryopolymerization temperature on the cryogel’s compression moduli. The compression moduli decreased proportionally to the temperature across the three types of cryogels studied. As shown in video S1, HAGM and HA-*co*-Gelatin cryogels were elastic, soft, sponge-like materials that could withstand large deformations and be compressed without any apparent mechanical damage. However, pure MA-gelatin cryogels were quite weak and easily broken, independently of their fabrication temperatures. Stress-strain curves revealed that HAGM cryogels fabricated at −20 and −50 °C have the largest moduli (Figure 3B). HAGM and HA-*co*-Gelatin cryogels fabricated at different subzero temperatures could withstand up to 90% compression strain without any permanent deformation or mechanical destruction, demonstrating their potential as a biomaterial for minimally invasive delivery. However, all MA-gelatin cryogels had modulus values below 1 kPa and performed poorly during compression testing.

Cryogels were also subjected to swelling ratio measurements. As shown in Figure 3C, HAGM cryogels had the highest swelling ratios (Q_M_ = 50 to 60). In comparison, MA-gelatin cryogels exhibited a lower swelling ratio (Q_M_ = 20 to 30). As expected, HA-*co*-Gelatin cryogels exhibited swelling ratio values in between (Q_M_ = 28 to 40). Across the different polymer formulations, the swelling ratios and fabrication temperatures were proportional. The lower the temperature was, the smaller the swelling ratio that was observed. Additionally, the degree of interconnectivity, which is another physical property was evaluated for each individual cryogel fabricated in this study and is shown in Figure 3D. Regardless of the polymer formulation or cryopolymerization temperature, cryogels exhibited a high degree of interconnectivity (~80%).

**Figure 4:**
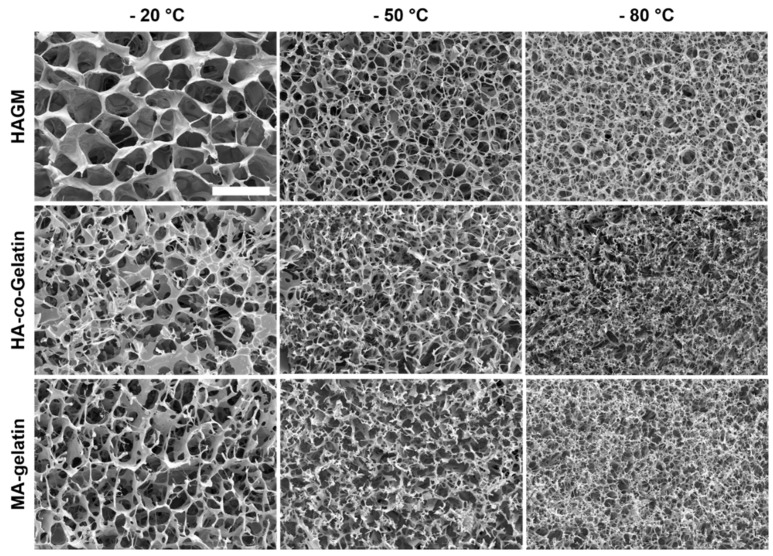
Macrostructure characterization. Scanning electron microscopy images of HAGM, MA-gelatin, and HA-*co*-Gelatin cryogels fabricated at −20, −50, and −80 °C for 15 h (scale bar = 100 µm).

As shown in Figure 4, all the investigated cryogels displayed an interconnected macroporous structure with thick polymer walls. However, the polymerization temperature, as well as the polymer formulation, influence the cryogel macrostructure. The polymerization temperature had a significant effect on the average pore size of the cryogels. Lowering the temperature from −20 to −80 °C led to the fabrication of cryogels with smaller pore sizes, more likely due to the formation of smaller ice crystals during the ice nucleation process. For HAGM cryogels, SEM images revealed average pore sizes of 70 ± 10, 40 ± 10 and 30 ± 5 µm upon cryopolymerization at −20, −50 and −80 °C, respectively. The average pore sizes of MA-gelatin cryogels polymerized at −20, −50 and −80 °C, were 80 ± 10, 40 ± 10 and 20 ± 5 µm, respectively. When combining the two polymers, the average pore sizes was 60 ± 10, 50 ± 10 and 20 ± 5 µm upon cryopolymerization at −20, −50 and −80 °C, respectively.

### 3.4. Injectability of Cryogels

The ability of cuboid-shaped cryogels to pass through a conventional 16-gauge needle and to regain their original shapes once delivered was also evaluated (Video S1). The large volumetric change of cryogels across the three different polymer formulations was presumably caused by a reversible collapse of their highly interconnected macropores. HAGM and HA-*co*-Gelatin cryogels fabricated at the three subzero temperatures successfully passed through the needle with no visible fracture or damage. After injection, the deformed cryogels rapidly returned to their original, undeformed configuration (Video S1), as surrounding water was reabsorbed into the gels. However, MA-gelatin cryogels were more mechanically fragile and fractured during syringe injection.

### 3.5. Biological Properties of Cryogels

**Figure 5:**
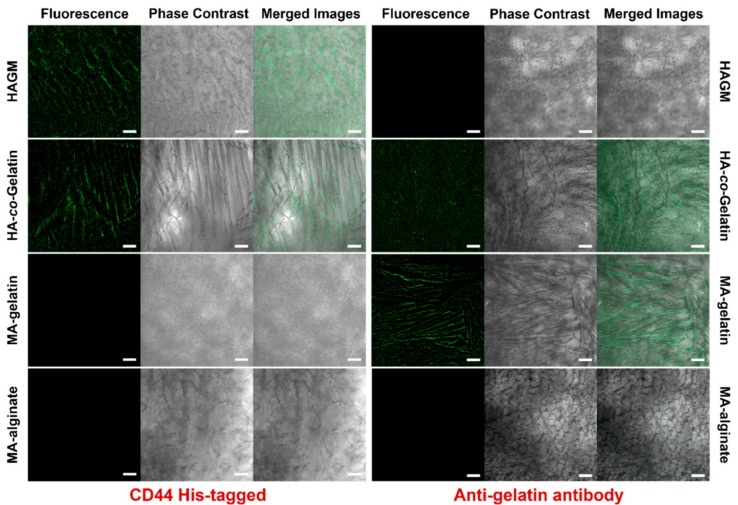
Evaluation of biological properties of cryogels. Immunostaining of HAGM, MA-gelatin, HA-*co*-Gelatin, and MA-alginate cryogels using human CD44 His-tagged receptor and anti-gelatin antibody. CD44 binding was depicted with an Alexa Fluor 488-conjugated 6x-His Tag monoclonal antibody (green), and anti-gelatin antibody binding was detected with a FITC-conjugated anti-rabbit IgG (green) (scale bar = 100 µm).

Recent studies have demonstrated that both HA and gelatin have unique intrinsic properties allowing cell attachment and spreading via CD44 and integrin receptors, respectively [68,70]. Thus, we assessed the conservation of these biological properties after methacrylation and polymerization via immunofluorescence assays using either CD44 receptors or anti-gelatin antibodies. As depicted in Figure 5, confocal images highlighted that the polymer walls of HAGM cryogels were specifically stained with the fluorescent anti-CD44 antibody. This set of data indicates that the chemically modified and crosslinked HA has unchanged biological behavior and is able to bind strongly to CD44 protein. In addition, FITC-labeled anti-gelatin antibody was used to evaluate the biological properties of MA-gelatin. Similar to HA, Figure 5 confirms that the chemically modified and crosslinked gelatin interacted efficiently and specifically with the fluorescent anti-gelatin antibody. These data suggest retention of the inherent biological properties of gelatin. Both antibody-labeling experiments were also performed on HA-*co*-Gelatin. As shown in Figure 5, these cryogels, positive for both antibodies, exhibit combined biologically active sites from both HA and gelatin. MA-alginate cryogels, used as negative control, did not interact with either HA-specific receptors or anti-gelatin specific antibodies. These results clearly confirm that HA and gelatin, individually or in combination, retained their intrinsic biological properties following chemical modification and covalent crosslinking.

### 3.6. In Vitro Cell Attachment, Survival, and Spreading

**Figure 6:**
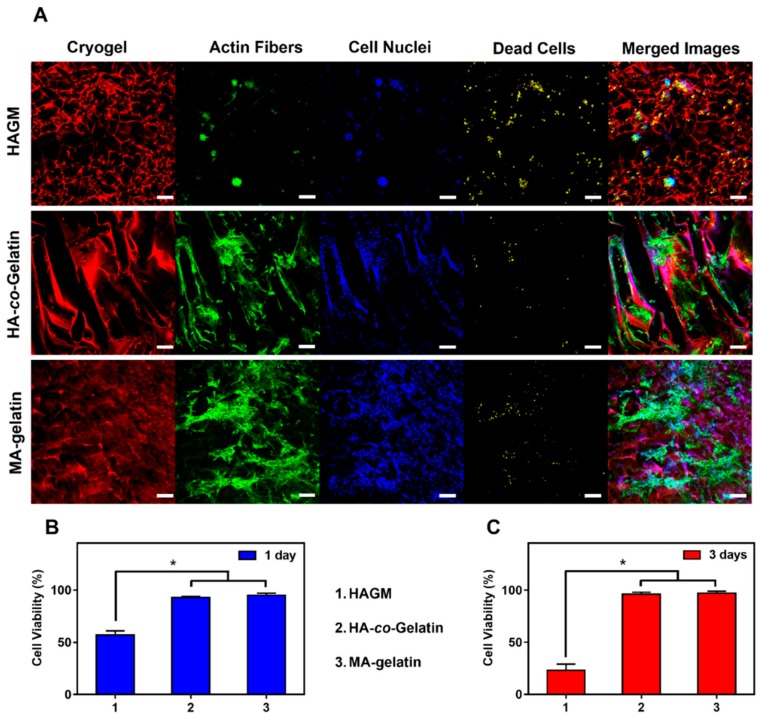
In vitro quantitative and qualitative evaluation of cell viability. (**A**) Confocal images of 2 × 10^5^ mouse 3T3 embryonic fibroblast cells cultured for three days within HAGM, MA-gelatin, and HA-*co*-Gelatin cryogels. The polymer walls of cryogels are labeled with Rhodamine (red), cell nuclei with DAPI (blue), dead cells with far-red fixable dead cell staining (yellow), and cytoskeleton with Alexa Fluor 488-phalloidin (green). Viability of 3T3 embryonic fibroblast cells on the three types of cryogels after (**B**) one day and (**C**) three days’ incubation. All cryogels were fabricated at −20 °C. Values represent mean and SD (*n* = 5, scale bar = 100 µm). Viability data were analyzed using ANOVA (* *p* < 0.05).

Next, we evaluated the suitability of these cryogels as substrates for cell attachment and spreading. Cryogels were partially dehydrated, then seeded with 2 × 10^5^ of 3T3 cells. Absorption of the cell suspension through the open-pore structure of cryogels enabled uniform cell seeding throughout the construct. Cells were caged and entrapped in gel pores. Cryogels composed of MA-gelatin provided an appropriate microenvironment for cell attachment and survival leading to 95% of viability after one day and three days of incubation (Figure 6). As anticipated, the results were different with HAGM cryogels. Cells did not strongly adhere to the scaffolds and as a result formed cellular aggregates leading to poor cell engraftment (Figure 6A) and poor viability, dropping from 55% to 25% after one day and three days of incubation (Figure 6B,C). On the other hand, cells attached successfully on the polymer walls of both MA-gelatin and HA-*co*-Gelatin cryogels within a day (Figure S2). Longer incubation time (three days) led to the formation of a monolayer of cells on the surface of both MA-gelatin and HA-*co*-Gelatin cryogels, suggesting cell spreading (Figure 6A).

### 3.7. In Vitro Stimulation of BMDCs

**Figure 7:**
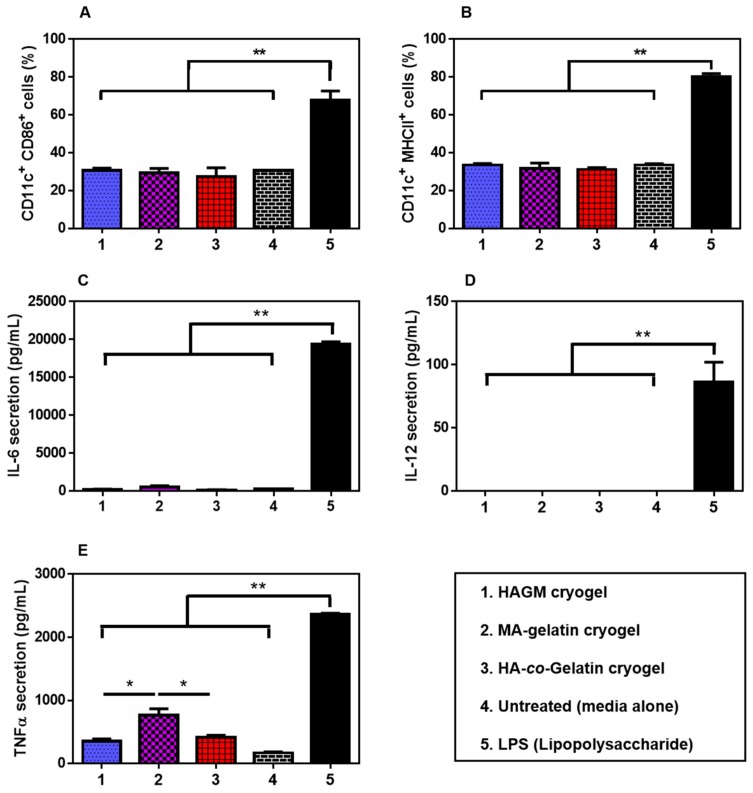
In vitro activation of bone marrow-derived dendritic cells (BMDCs). Fraction of activated (**A**) CD11c^+^CD86^+^ and (**B**) CD11c^+^MHCII^+^ BMDCs stimulated after exposure to HAGM, HA-*co*-Gelatin, and MA-gelatin cryogels for 24 h. Concentrations of (**C**) IL-6; (**D**) IL-12; and (**E**) TNF-α from the cell culture supernatant when BMDCs were exposed to HAGM, HA-*co*-Gelatin, and MA-gelatin cryogels for 24 h. Lipopolysaccharide (LPS) at 100 ng/mL was used as a positive control and cryogel-free medium as a negative control (untreated). Values represent mean and SD (*n* = 5). Data were analyzed using ANOVA (* *p* < 0.05).

Dendritic cells play a critical role in the regulation of human adaptive immunity, as they are known to secrete various proinflammatory cytokines upon maturation, inducing immune responses [71]. HAGM, MA-gelatin, and HA-*co*-Gelatin cryogels were assessed for their immunogenicity with BMDCs. The fraction of activated CD11c^+^CD86^+^ and CD11c^+^MHCII^+^ BMDCs was detected by immunostaining in conjunction with flow cytometry after 24 h culture with the cryogels. All the samples, including the negative control (media alone), HAGM cryogels, MA-gelatin cryogels, and HA-*co*-Gelatin cryogels induced low-expression profiles of CD86 and MHCII receptors when compared to LPS, our positive control (Figure 7A,B). ELISA analysis of IL-6, IL-12, and TNF-α secretion levels in the supernatant were consistent with the flow cytometry results (Figure 7C–E). After 24 h culture with the cryogels, minimal proinflammatory cytokines were secreted (Figure 7C). Furthermore, a low secretion of TNF-α was induced by HAGM and HA-*co*-Gelatin cryogels. However, secretion of TNF-α was slightly increased for MA-gelatin cryogels, more likely due to partial degradation and release of gel-fragments (Figure 7D). Overall, these results suggest that the three different cryogels did not significantly activate BMDCs.

## 4. Discussion

Three-dimensional, highly porous scaffolds with an interconnected macroporous structure can be very useful for tissue-engineering applications [15]. A scaffold is intended to provide biomechanical support imitating native tissues, serving as an ECM analog [72]. Ideally, scaffolds should promote cell–material interactions, support the movement of nutrients and waste, possess good biocompatibility, and have appropriate mechanical properties [49]. However, their placement in the body remains a challenge and often requires performing an invasive surgical procedure [73]. Previously developed injectable strategies that rely on in situ gelation post-injection often face limitations such as inflammation or poor biophysical properties [74]. Development of shape-memory scaffolds, able to undergo syringe injection and full recovery at the injection site, while having tunable physicochemical properties, could facilitate the development of new minimally invasive therapies and may improve current tissue-engineering approaches [18,20,49].

In this study, we fabricated injectable cryogels from HA, gelatin, and their mixture. These cryogels were fabricated at different subzero temperatures to evaluate the effect of fabrication temperature on the physical properties, biocompatibility, and biological properties of cryogels. Through a combination of physical and imaging characterizations, cell viability, and biological properties evaluation, we showed that HA-*co*-Gelatin cryogels have significantly improved properties when compared to cryogels made from each polymer individually. HA-*co*-Gelatin cryogels combined the favorable inherent properties of each biopolymer while retaining shape-memory properties and syringe injectability (Video S1). Based on these data, we propose these naturally derived and minimally invasive cryogels as a promising 3D platform for tissue reconstruction.

We observed that decreasing the fabrication temperature can lead to the formation of smaller ice crystals, which results in the formation of smaller pores [33]. Unlike other minimally invasive delivery approaches that require polymer injection and in situ gelation, gels made using cryopolymerization are able to retain predefined geometries, architecture, and porosity posti-njection [62,63,65,75,76,77,78]. Similarly, physical properties of cryogels were dependent on the fabrication temperature. Decreasing the fabrication temperature impacted the swelling ratio and compression moduli of cryogels for both types of polymers and their mixture, likely due to a higher freezing rate [33,79]. Rapid freezing of the polymer solution may result in surpercooling of water which could lead to smaller and irregular ice crystal formation, the fabrication of weaker cryogels, and lower swelling ratios. Cryogels fabricated at the lowest temperatures (−50 and −80 °C) tend to be less flexible and therefore more susceptible to breakage during compression and injection.

Our results further confirmed that HA, although a naturally derived and non-immunogenic polymer, which can be formed into an injectable cryogel, has poor cell adhesion properties [31]. Previous reports on incorporating different types of polymers or peptides with HA showed improved bioadhesive properties [32,35,48,49,80]. However, the addition of peptides as a comonomer into the cryogels not only adds an extra step to the fabrication process, but it can also diminish the mechanical stability of cryogels due to a decrease in crosslinking density [81,82]. Reciprocally, MA-gelatin cryogels had poor mechanical properties, independent of their fabrication temperature, which led to the breakage of these cryogels during injection that could be rescued by combination with HAGM [83].

Evaluation of biological properties of the cryogels fabricated in this study revealed that HA-*co*-Gelatin cryogels are biologically active (bioadhesion and CD44-mediated HA recognition) towards gelatin and HA despite their chemical modification and covalent crosslinking. Addition of gelatin to HA cryogels improved the viability of 3T3 fibroblast cells without the need for additional peptide grafting. Furthermore, in vitro stimulation of BMDCs when exposed to HA, gelatin, and HA-*co*-Gelatin cryogels indicated that these cryogels have great potential to be utilized safely.

Although in our study HA-*co*-Gelatin cryogels exhibited improved properties, we only studied a single ratio (50:50) of the two polymers. Further tuning of biophysical properties to match specific tissues might be possible by changing the ratio of biopolymers to better recapitulate tissue-specific microenvironments. Similarly, other biopolymers could potentially be added to generate more complex cryogel composition with improved biological properties. Such cryogels would require more in vitro (using various cell types and coculture models) and in vivo (animal models) studies to better characterize the biocompatibility profile of the generated scaffolds. Previous studies have shown that proteins and drugs can be entrapped within the dense polymer walls of cryogels and be released subsequently [20]. However, the high surface-to-volume ratio of cryogels needs to be considered when designing such drug-delivery systems. Even with unique compressible properties, cryogels have a size limitation in order to be successfully injected through conventional needles. To date, cuboid-shaped cryogels as large as 8 mm × 8 mm × 1 mm have been injected through a 16-gauge needle [35]. However, when needed, larger cryogels could potentially be injected through a catheter for specific tissue-engineering applications.

## 5. Conclusions

HAGM, MA-gelatin, and HA-*co*-Gelatin cryogels were successfully fabricated under different conditions. The results of chemical characterization showed that the polymerization can be completed at temperatures as low as −80 °C after 15 h reaction. Furthermore, we obtained greater insights into the effects of fabrication temperature on physical properties as well as macrostructure of the cryogels. Specifically, we demonstrated that the physical properties of HA-*co*-Gelatin cryogels can be modulated by changing the temperature during cryopolymerization. Additionally, a very important and promising result of this study is that HA-*co*-Gelatin cryogels retain suitable mechanical properties, bioactivity (cell adhesion and CD44-mediated HA recognition), and injectability. These cryogels rapidly regained their original shape and size once injected. Furthermore, we observed neither significant proinflammatory secretion of cytokines nor activation of BMDC when exposed to HA-*co*-Gelatin cryogels. Taken all together, these properties position HA-*co*-Gelatin cryogels as a potential platform for tissue-engineering efforts.

## Supplementary Materials

The followings are available online at http://www.mdpi.com/1996-1944/11/8/1374/s1, Figure S1: Chemical characterization of polymers by ^1^H NMR. ^1^H NMR spectra of uncross-linked HAGM (bottom), MA-gelatin (center), and HA-*co*-Gelatin (top) in D_2_O. Figure S2: In vitro quantitative and qualitative evaluation of cell viability. Confocal microscopy images of 2 × 10^5^ mouse 3T3 embryonic fibroblast cells cultured for one day within the HAGM, MA-gelatin, and HA-*co*-Gelatin cryogels. The cryogel wall is labeled with Rhodamine (red), cell nuclei with DAPI (blue), dead cells with Far-Red Fixable Dead Cell Stain Kit (red), and cytoskeleton with Alexa Fluor 488-phalloidin (green) (scale bar = 100 µm). Video S1: Injection of 4% (*w*/*v*) HAGM cryogel, 4% (*w*/*v*) MA-gelatin cryogel, and 4% (*w*/*v*) HA-*co*-Gelatin cryogels through a 16-gauge needle.
