# Supplementary material for: Injectable Hyaluronic Acid-co-Gelatin Cryogels for Tissue-Engineering Applications
Source: Materials (Basel). 2018 Aug 7;11(8):1374. doi: 10.3390/ma11081374 (PMC6119876; doi:10.3390/ma11081374)
Supplement: Supplementary file 1 [file materials-11-01374-s001.pdf]

Supplementary Information

Injectable Hyaluronic Acid-*co*-Gelatin Cryogels for Tissue Engineering Applications

Mahboobeh Rezaeeyazdi [1], Thibault Colombani [1], Adnan Memic [1,2] and Sidi. A. Bencherif [1,3,4,5] *

[1] Department of Chemical Engineering, Northeastern University, Boston, USA; rezaeeyazdi.m@husky.neu.edu (M.R.); t.colombani@northeastern.edu (T.C.)
[2] Center of Nanotechnology, King Abdulaziz University, Jeddah, Saudi Arabia; amemic@gmail.com
[3] Department of Bioengineering, Northeastern University, Boston, USA
[4] Harvard John A. Paulson School of Engineering and Applied Sciences, Harvard University, Cambridge, USA
[5] Biomechanics and Bioengineering (BMBI)- UTC CNRS UMR 7338, University of Technology of Compiègne, Compiègne, France
* Correspondence: s.bencherif@northeastern.edu; Tel.: +1-617-373-7103

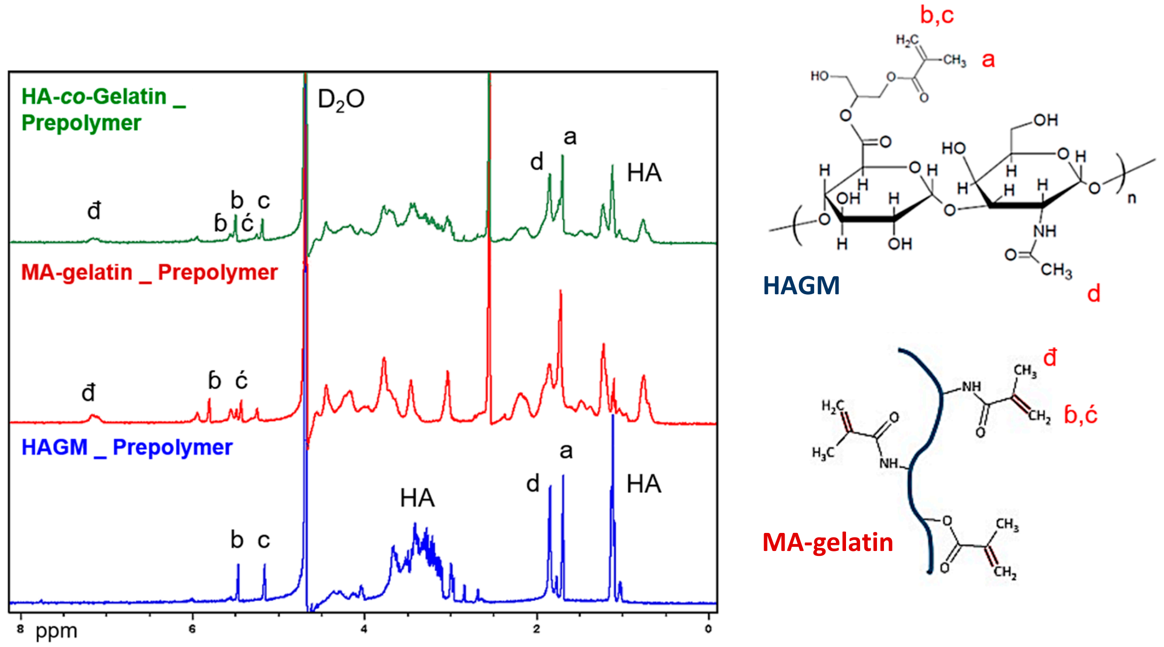

Figure S1. Chemical characterization of polymers by ¹H NMR. ¹H NMR spectra of uncross-linked HAGM (bottom), MA-gelatin (center), and HA-*co*-Gelatin (top) in D_2O.

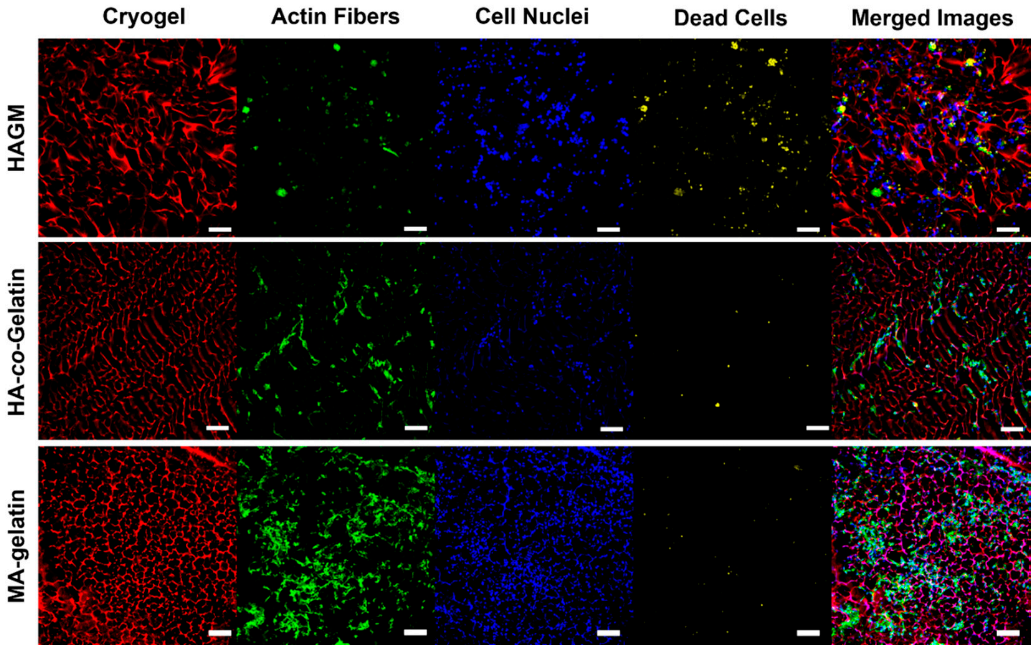

Figure S2. Qualitative evaluation of cell viability. Confocal microscopy images of 2 x 10^5 mouse 3T3 embryonic fibroblast cells cultured for 1 days in HAGM, MA-gelatin, and HA-*co*-Gelatin cryogels. The cryogel wall is labeled with Rhodamine (red), cell nuclei with DAPI (blue), dead cells with Far-Red Fixable Dead Cell Staining (yellow), and cytoskeleton with Alexa Fluor 488-phalloidin (green) (scale bar = 100 μm).

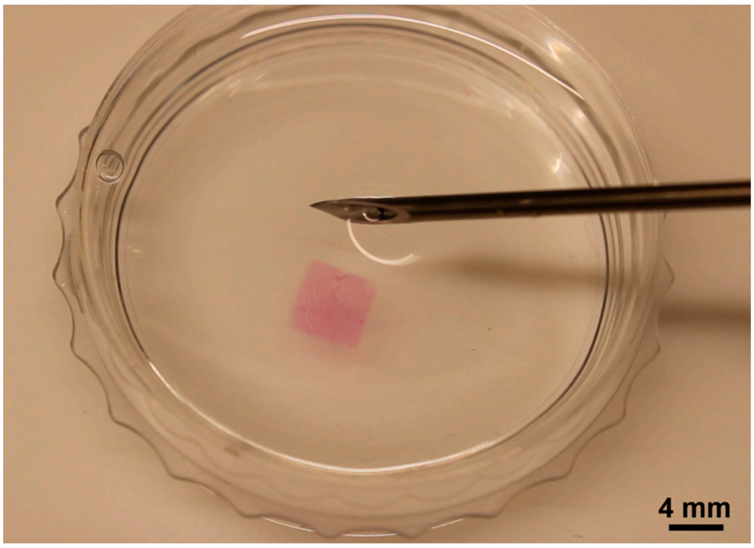

Video S1. Injection of 4% (w/v) HAGM cryogel, 4% (w/v) MA-gelatin cryogel, and 4% (w/v) HA-*co*-Gelatin (50:50) cryogels through an 16G needle.
